# Childhood Maltreatment Is Associated with Fire-Related Attitudes: The Role of Environmental Identity in a Portuguese Community Sample

**DOI:** 10.3390/bs16101730

**Published:** 2026-09-23

**Authors:** Paulo Ferrajão, Inês Lorenzo, Jorge Monge, Júlia Fonte, Amadeu Quelhas Martins

**Affiliations:** 1 Faculdade de Ciências da Saúde, Universidade Europeia, 1500-210 Lisbon, Portugal; 50048273@europeia.pt (I.L.); 50048680@europeia.pt (J.M.); julia.fonte@ext.universidadeeuropeia.pt (J.F.); amadeu.martins@universidadeeuropeia.pt (A.Q.M.); 2 CIDESD—Research Center in Sports Sciences, Health and Human Development, 6200-001 Covilhã, Portugal; 3 UNIDCOM/IADE, Unidade de Investigação em Design e Comunicação, 1886-502 Moscavide, Portugal

**Keywords:** deliberate fire-setting, adverse childhood experiences, environmental identity, nature protection, residential context

## Abstract

Deliberate fire-setting represents a significant environmental and public safety concern, yet research has primarily focused on identified firesetters and forensic or clinical populations. This study examined associations between childhood maltreatment and self-reported fire-related attitudes and tendencies in a Portuguese community sample, considering environmental identity as a potential indirect pathway and residential context in these associations. A cross-sectional online survey was conducted among 5077 Portuguese adults using self-report measures of childhood maltreatment, environmental identity, and fire-related attitudes and tendencies. Structural equation modeling and bootstrapped indirect-effect analyses were complemented by multigroup path analyses across rural, suburban, and urban settings. Physical abuse, sexual abuse, emotional neglect, and physical neglect were directly associated with higher levels of fire-related attitudes and tendencies. Significant indirect associations through connectedness with nature were observed for emotional abuse, sexual abuse, emotional neglect, and physical neglect, whereas protection of nature was involved in a specific indirect association with emotional neglect. Structural associations were largely comparable across residential contexts, despite modest group differences in construct levels. These findings suggest that developmental adversity and psychological relationships with nature are relevant correlates of fire-related attitudes and tendencies, while causal interpretations and inferences about actual fire-setting behavior remain unwarranted.

## 1. Introduction

### 1.1. Deliberate Fire-Setting: Definitions, Prevalence, and Risk Factors

Fire has long played a fundamental role in human societies, providing essential sources of heat and energy. However, anthropogenic climate change has contributed to an increase in the frequency and intensity of extreme weather events, thereby creating environmental conditions conducive to the ignition and spread of wildfires (Gannon et al., 2012; Wehner et al., 2022). Although wildfires can result from both natural and human causes, a proportion are deliberately ignited, constituting a serious and potentially preventable threat to environmental and public safety. Deliberately ignited fires can threaten communities, cause extensive damage to ecosystems and biodiversity, and generate substantial economic costs. These impacts highlight the need to better understand the psychological and contextual factors underlying deliberate fire-setting and to develop more effective strategies for prevention and mitigation (Dadswell et al., 2023; Johnston & Tyler, 2025).

Portugal’s Mediterranean climate and geographic location are important determinants of wildfire occurrence and spread. The interaction between the Azores High, the thermal low-pressure system over the Iberian Peninsula, and the influx of hot, dry air from Continental Europe and North Africa creates meteorological conditions that favor fire ignition and rapid wildfire propagation (Couto et al., 2022; Ermitão et al., 2023). Between 2012 and 2020, Portugal recorded an average of approximately 8841 urban fires annually (Bispo et al., 2023). Together with high population density and complex urban infrastructure, these events impose substantial economic costs through firefighting operations, property damage, employment losses, and adverse effects on tourism, while also posing significant risks to human life. Importantly, approximately 99.4% of forest fires in Portugal have been attributed to human causes, with 75% classified as intentional and 25% resulting from negligence (Calheiros et al., 2022). These findings underscore the societal relevance of human involvement in wildfire occurrence and support examining psychological factors associated with fire-related attitudes and tendencies. The present study does not assume deliberate fire-setting among community participants; rather, it examines individual differences in self-reported fire-related attitudes and tendencies, ranging from normative interest to potentially maladaptive orientations. This approach may inform understanding of factors relevant to deliberate fire-setting without equating self-reported tendencies with actual behavior.

Estimating both the number and characteristics of individuals responsible for deliberately set wildfires remains challenging, and many perpetrators are never identified (Johnston & Tyler, 2025). Despite the substantial societal and environmental consequences associated with deliberate fire-setting, research on this phenomenon remains comparatively limited, particularly in adult populations (Johnston & Tyler, 2025). Consequently, much of the evidence linking developmental adversity, psychological characteristics, and deliberate fire-setting is based on theoretical models and studies of identified fire-setters rather than on population-based research. This limitation highlights the need for research examining psychological correlates of fire-related attitudes and tendencies in community samples.

Previous research indicates that deliberate firesetting is more prevalent among men than women. In a nationally representative U.S. sample, Blanco et al. (2010) reported a lifetime prevalence of fire-setting of 1.7% among men, compared with 0.4% among women. Earlier clinical research likewise documented a marked male predominance among individuals who engage in fire-setting (Bourget & Bradford, 1989). Sex differences have also been reported in the characteristics and correlates of fire-setting, with evidence suggesting that women who engage in fire-setting may exhibit somewhat different psychological and behavioral profiles from their male counterparts (Hoertel et al., 2011; Nanayakkara et al., 2020; Kitson-Boyce et al., 2025).

However, these findings primarily concern individuals with a history of actual fire-setting and therefore should not be directly generalized to the present community sample. The relatively high proportion of women in the present study is relevant not because the sample is expected to reflect the sex distribution of individuals who engage in fire-setting, but because the study examines individual differences in self-reported fire-related attitudes and tendencies in the general population. From this perspective, examining gender is potentially informative regarding whether factors associated with fire-related attitudes and tendencies operate similarly across women and men, even in the absence of documented fire-setting behavior. Accordingly, sex was considered an important demographic characteristic in interpreting the present findings, while recognizing that the composition of the community sample does not represent the sex distribution of individuals who engage in deliberate fire-setting.

Importantly, deliberate fire-setting encompasses heterogeneous phenomena and should not be regarded as synonymous with either isolated fire-setting incidents or clinically defined pyromania. Isolated fire-setting may occur as a consequence of curiosity or experimentation, particularly during childhood and adolescence, and does not necessarily develop into persistent or destructive patterns of fire-setting (Brereton et al., 2020). Individuals involved in isolated fire-setting incidents tend to be young, curious, socially naïve, inexperienced in handling flammable materials, impulsive, and prone to resentment, although most do not engage in repeated fire-setting (Palermo, 2015).

Pyromania is a psychiatric disorder characterized by recurrent episodes of deliberate fire-setting, accompanied by increasing tension or emotional arousal before the act and a sense of relief or gratification afterwards (Wehner et al., 2022). Arson, by comparison, refers to the intentional and unlawful setting of fires and may be motivated by a range of factors, including revenge, financial gain, concealment of criminal activity, or, less commonly, pyromania (Merrick et al., 2013). Although both arson and fire-setting associated with pyromania involve deliberate ignition, arson is typically characterized by an external motive, whereas pyromania involves recurrent fire-setting driven primarily by an internal impulse rather than by external incentives (Palermo, 2015). These distinctions are particularly important when interpreting self-reported fire-related attitudes and tendencies, which may reflect a broader continuum of fire-related cognition and propensity rather than verified criminal behavior.

Individuals identified as engaging in arson have consistently been reported to exhibit elevated rates of psychiatric disorders, social isolation, criminal offending, and family histories of antisocial behavior (Merrick et al., 2013; Palermo, 2015). Poor impulse control, aggression, and cruelty toward others have also been identified as correlates of fire-setting (Brereton et al., 2020). Evidence from child and adolescent populations further suggests that deliberate fire-setting is associated with psychopathology, antisocial and externalizing behaviors, social and interpersonal difficulties, and adverse developmental experiences, including family dysfunction, exposure to violence, and trauma (Brereton et al., 2020). However, the extent to which these findings generalize to adults and to non-forensic populations remains unclear.

Evidence suggests that childhood maltreatment is associated with an increased likelihood of deliberate fire-setting through heightened emotional and behavioral difficulties (Root et al., 2008). Childhood abuse and neglect can disrupt the development of emotion regulation skills and thereby increase the vulnerability to difficulties in expressing negative emotions, such as anger and anxiety, and to maladaptive externalizing behaviors, including deliberate fire-setting (Root et al., 2008). Such behaviors may represent maladaptive coping strategies in response to environmental stressors and attempts to communicate or alleviate emotional distress (Root et al., 2008). Moreover, childhood trauma, including physical or sexual abuse and the loss of a family member, has been associated with long-term socioemotional and behavioral consequences that may influence a range of later outcomes, including environmentally relevant behaviors (Raja & Carrico, 2022).

Childhood maltreatment has been associated with a greater likelihood of deliberate fire-setting, potentially through its links with emotional and behavioral difficulties (Root et al., 2008). Childhood abuse and neglect may disrupt the development of emotion regulation skills, increasing vulnerability to difficulties in managing negative emotions, such as anger and anxiety, and to maladaptive externalizing behaviors (Root et al., 2008). Such behaviors may function as maladaptive responses to environmental stressors or as attempts to communicate or alleviate emotional distress (Root et al., 2008). More broadly, childhood trauma, including physical or sexual abuse and the loss of a family member, has been associated with long-term socioemotional and behavioral consequences that may influence a range of later outcomes, including environmentally relevant attitudes and behaviors (Raja & Carrico, 2022). These findings provide a developmental basis for examining whether childhood maltreatment is associated with adult fire-related attitudes and tendencies.

Interest in fire has also been consistently identified as an important psychological correlate of deliberate fire-setting (Dadswell et al., 2023; Wehner et al., 2022). Although much of the existing literature has examined fire-related behaviors in younger populations, engagement with fire is often conceptualized along a continuum ranging from normative curiosity to maladaptive and intentional fire-setting, depending on individual and environmental risk factors (Dadswell et al., 2023). Fire interest, defined as a general fascination with fire and fire-setting, has been associated with a greater likelihood of deliberate fire-setting and greater behavioral severity, highlighting its relevance as a potential correlate of intentional fire-related offending (Dadswell et al., 2023; Wehner et al., 2022).

One influential framework that identifies fire interest as a key factor underlying deliberate fire-setting is the Multi-Trajectory Theory of Adult Firesetting (M-TTAF). The M-TTAF proposes that deliberate fire-setting emerges from the interaction of psychological vulnerabilities, developmental and sociocultural influences, situational factors, and social learning processes (Wehner et al., 2022). According to this model, four broad domains of psychological vulnerability may contribute to deliberate fire-setting: inappropriate interest in and beliefs about fire, offense-supportive cognitions, deficits in self- and emotion regulation, and interpersonal and communication difficulties (Johnston & Tyler, 2025). These vulnerabilities are shaped by biological, developmental, and environmental experiences and may be maintained through internal and external reinforcement processes, as well as through post-fire emotional and cognitive responses (Johnston & Tyler, 2025).

Within the M-TTAF, the fire-interest trajectory is characterized by fire-supportive attitudes and beliefs, including perceptions of control over fire (Wehner et al., 2022). Stress, boredom, and excitement are proposed as important motivational states, with deliberate fire-setting potentially functioning as a maladaptive strategy for regulating these experiences while providing emotional or sensory gratification (Wehner et al., 2022). The model also identifies sensation seeking, defined as a preference for novel and intense experiences accompanied by a willingness to take risks, as a potential contributor to fire-setting. Indeed, susceptibility to boredom has been associated with deliberate fire-setting among adults, suggesting that fire-related tendencies may be linked to attempts to alleviate under-stimulation (Wehner et al., 2022). Accordingly, assessing fire interest and related attitudes may be relevant to risk assessment and may inform prevention and early intervention strategies (Dadswell et al., 2023).

An ecological risk model further proposes that deliberate fire-setting is influenced by the interaction of individual, familial, and environmental risk factors (Fritzon et al., 2011). Predisposing factors include cognitive, behavioral, and motivational characteristics, together with parental influences, family stressors, exposure to fire-related models during childhood, access to ignition materials, and previous fire-setting by siblings or peers (Fritzon et al., 2011). At the individual level, limited social and emotional regulation skills, difficulties with anger management, covert behaviors, and heightened interest in fire have been identified as relevant risk factors, irrespective of the presence of mental disorders (Fritzon et al., 2011).

Individuals identified as engaging in deliberate fire-setting have also reported higher levels of childhood adversity, including harsh or abusive parenting and greater parental psychological dysfunction. These findings suggest that family environments characterized by inadequate supervision, parental disengagement, parental mental health difficulties, and exposure to stressful life events may be associated with greater vulnerability to fire-setting (Fritzon et al., 2011). Nevertheless, the model also acknowledges that fire-setting may contribute to family dysfunction, highlighting the potentially reciprocal nature of these associations (Fritzon et al., 2011).

### 1.2. Environmental Identity, Connectedness with Nature, and Nature-Protective Attitudes

Beyond individual and developmental risk factors, environmental psychology offers additional constructs that may contribute to understanding fire-related attitudes and tendencies. In particular, environmental identity has emerged as an important construct for explaining individual differences in how people perceive, value, and interact with the natural environment. Environmental identity refers to the extent to which nature is incorporated into an individual’s self-concept, reflecting a sense of connection to the nonhuman world grounded in personal experiences, emotional attachment, and identification with the natural environment (Clayton, 2003; Schultz & Tabanico, 2007). As a relatively stable aspect of identity, it may shape cognitive, emotional, and behavioral responses toward nature and has been consistently associated with stronger environmental concern, a greater perceived moral obligation to protect the environment, and greater engagement in pro-environmental behaviors (Clayton, 2003; Schultz & Tabanico, 2007). Consequently, environmental identity may provide a useful framework for understanding not only behaviors intended to protect the natural environment but also attitudes and tendencies associated with environmentally destructive actions.

Environmental identity shares characteristics with other forms of collective identity in that it fosters a sense of connection and belonging that becomes integrated into the individual’s self-concept (Schultz & Tabanico, 2007). As a central aspect of identity, it may influence how individuals perceive themselves in relation to the natural environment, shaping self-reflection, values, and behavioral tendencies (Clayton, 2003). The development of environmental identity is influenced by direct experiences with nature, with greater exposure to natural environments generally associated with stronger feelings of environmental connectedness (Mena-García et al., 2020). However, environmental identity is not formed solely through direct interactions with nature; it is also shaped by the social and cultural meanings attributed to environmental issues, including cultural values and worldviews (Clayton, 2003).

Building on this conceptualization, environmental identity has been consistently associated with individuals’ attitudes, values, and behaviors toward the natural environment. By incorporating nature into the self-concept, environmental identity may influence how individuals interpret their relationship with the environment and guide behavioral responses that either support or undermine environmental protection (Clayton, 2003; Schultz & Tabanico, 2007). Individuals with stronger environmental identities tend to demonstrate greater environmental concern, a stronger sense of moral obligation toward nature, and greater engagement in pro-environmental behaviors, including public-sphere actions, such as environmental activism and civic engagement, and private-sphere practices, such as recycling and sustainable consumption (Anderson & Krettenauer, 2021; Veselý et al., 2021). The development and expression of environmental identity are further shaped by experiences with natural environments, as frequent and meaningful contact with nature may strengthen feelings of connectedness and identification with the nonhuman world (Mena-García et al., 2020).

Although environmental identity has predominantly been examined in relation to behaviors that protect the environment, its relevance may also extend to understanding behaviors that directly harm natural ecosystems. As mentioned above, environmental identity refers to the extent to which individuals perceive themselves as connected to the natural environment and incorporate nature into their self-concept, influencing environmental attitudes and behaviors (Clayton, 2003; Clayton & Opotow, 2003). Research has consistently shown that stronger environmental identities are associated with greater engagement in pro-environmental behaviors, such as conservation actions and reduced environmental impact (Whitmarsh & O’Neill, 2010; Gatersleben et al., 2014).

Although environmental identity has predominantly been examined in relation to behaviors that protect the environment, its relevance may also extend to attitudes and tendencies associated with behaviors that harm natural ecosystems. Environmental identity reflects the extent to which individuals perceive themselves as connected to the natural environment and incorporate nature into their self-concept, thereby influencing environmental attitudes and behavioral tendencies (Clayton, 2003; Clayton & Opotow, 2003). Research has consistently shown that stronger environmental identities are associated with greater engagement in pro-environmental behaviors, such as conservation actions and reduced environmental impact (Gatersleben et al., 2014; Whitmarsh & O’Neill, 2010).

Deliberate fire-setting can have substantial and potentially irreversible consequences for natural ecosystems. Examining environmental identity in relation to self-reported attitudes and tendencies toward deliberate fire-setting may therefore provide novel insights into psychological processes associated with environmentally harmful behavior (Nisbet et al., 2009; Schultz, 2001). This perspective may contribute to expanding current models of human–nature relationships beyond their predominant focus on environmental protection and toward a broader understanding of how self–nature relationships may be associated with both protective and destructive environmental orientations.

Moral concern for the environment does not necessarily translate into environmentally responsible behavior. This discrepancy, commonly referred to as the value–action or judgment–action gap, reflects the influence of contextual constraints, perceived costs, and competing motivations on the enactment of environmental values (Ajzen, 1991; Kollmuss & Agyeman, 2002). Personal norms, defined as an individual’s perceived moral obligation to engage in environmentally significant actions, have consistently been identified as important determinants of pro-environmental behavior within broader models of environmental decision-making (Bamberg & Möser, 2007; Stern, 2000). The activation of such norms is influenced by affective and empathic processes, which shape individuals’ willingness to consider the needs and vulnerability of others and of the natural world (Berenguer, 2007; Schultz, 2000). In particular, empathy toward nature has been associated with greater environmental concern, stronger nature-related identification, and greater motivation to engage in behaviors intended to protect ecosystems (Schultz, 2000; Tam, 2013).

However, moral and affective processes may also be relevant to understanding environmentally harmful attitudes and behaviors, as reduced moral engagement or psychological mechanisms that distance individuals from the consequences of their actions may facilitate actions that conflict with environmental values (Bandura, 2016). Examining these processes in the context of deliberate fire-setting may therefore provide new insights into psychological factors associated with environmentally destructive tendencies and contribute to a more comprehensive understanding of human–nature relationships beyond the traditional focus on environmental protection.

### 1.3. Residential Context and Experiences with Nature

Evidence suggests that not only the frequency but also the context of experiences with nature may shape environmental identity, connectedness with nature, and environmentally relevant attitudes and behaviors. Although individuals living in rural settings generally report stronger environmental identity and more positive environmental attitudes than their urban counterparts (Hinds & Sparks, 2008; Macias-Zambrano et al., 2024; Sierra-Barón et al., 2023), findings regarding connectedness with nature remain less consistent. Anderson and Krettenauer (2021) found no rural–urban differences in emotional connectedness with nature, whereas Sierra-Barón et al. (2023) suggested that associations among psychological health, connectedness to nature, environmental identity, and pro-environmental behavior may vary according to residential context. Together, these findings suggest that residential context may be relevant to the development and expression of environmental identity and environmentally relevant attitudes, highlighting its potential importance when examining self-reported tendencies toward environmentally destructive behavior.

Residential context may also represent an important intersection of relationships. Rural, suburban, and urban environments differ in opportunities for contact with nature, population density, social organization, access to environmental resources, and exposure to different forms of environmental stress (Duron-Ramos et al., 2020; Hartig et al., 2014; van den Berg et al., 2007). Rural, suburban, and urban environments differ in opportunities for contact with nature, population density, social organization, access to environmental resources, and exposure to different forms of environmental stress. These contextual differences may shape both environmental identity and the expression of fire-related attitudes and tendencies (Hinds & Sparks, 2009). However, whether associations between childhood maltreatment, environmental identity, and fire-related tendencies are equivalent across residential contexts remains insufficiently understood.

### 1.4. The Present Study

The present study examines the associations between different forms of childhood maltreatment and self-reported attitudes, interest, and tendencies related to deliberate fire-setting in a community sample. Importantly, the study does not assume that participants have engaged in deliberate fire-setting, nor does it assess verified fire-setting behavior. Rather, it focuses on individual differences in self-reported fire-related attitudes and tendencies that may range from normative curiosity or interest in fire to more maladaptive orientations toward deliberate fire-setting. Within this framework, we investigate whether developmental adversity is associated with such fire-related psychological tendencies and whether these associations may be indirectly related to individuals’ relationships with the natural environment.

Building on ecological and developmental perspectives, the study considers connectedness with nature and nature-protective attitudes as potential factors linking childhood experiences to fire-related attitudes and tendencies. Specifically, exposure to different forms of childhood maltreatment may be associated with individual differences in self-reported attitudes and tendencies toward deliberate fire-setting, consistent with evidence linking adverse developmental experiences to externalizing and maladaptive outcomes (Root et al., 2008; Fritzon et al., 2011). At the same time, positive and sustained interactions with natural environments during development have been associated with stronger connectedness with nature and greater engagement in behaviors aimed at protecting the natural environment (Rosa et al., 2018; Veselý et al., 2021). Accordingly, childhood maltreatment may also be associated with differences in individuals’ relationships with the natural environment, which may in turn be reflected in their environmental identity and in their self-reported fire-related attitudes and tendencies.

The present study further examines whether these patterns of association differ across rural, suburban, and urban residential contexts. By integrating developmental adversity, environmental connectedness, nature-protective attitudes, and self-reported fire-related tendencies within a single framework, the study seeks to extend understanding of psychological factors associated with deliberate fire-setting attitudes in the general population. In doing so, the study deliberately distinguishes between self-reported psychological attitudes or tendencies related to fire-setting and actual fire-setting behavior, and therefore does not interpret the observed associations as evidence that participants have engaged in deliberate fire-setting.

## 2. Materials and Methods

### 2.1. Sample and Study Design

The study sample comprised 5077 Portuguese adults aged 18 years and older, with a mean age of 33.75 years (SD = 14.33; range = 18–78). As presented in Table 1, the sample consisted predominantly of female participants. Regarding marital status, the majority of participants were single, while a substantial proportion reported being married or cohabiting. In terms of educational attainment, most participants reported having completed higher education, followed by 12 years of formal schooling, whereas only a small proportion had nine years of education or less. Regarding professional status, the majority of participants were either employed or students, whereas a smaller proportion reported combining work and study; only a minority of participants were unemployed or retired. As for residential setting, most participants reported residing in urban areas, followed by suburban settings, whereas only a minority lived in rural areas. Finally, most participants self-identified as belonging to the middle socioeconomic stratum, whereas smaller proportions reported upper-middle or lower socioeconomic status. Only a very small proportion classified themselves as belonging to a high socioeconomic stratum (see Table 1).

This study was part of a broader research project examining the psychological correlates of fire-setting behavior. Ethical approval was obtained from the Institutional Review Board of Universidade Europeia. Participants were recruited between March and May 2026 using a convenience sampling strategy through mailing lists and social media platforms, including Facebook, Instagram, LinkedIn, and WhatsApp. Participants were recruited between March and May 2026 using a convenience sampling strategy, with recruitment conducted through mailing lists and social media platforms, including Facebook, Instagram, LinkedIn, and WhatsApp. Eligibility criteria included being 18 years of age or older, having Portuguese as their first language, and having sufficient educational background to understand the questionnaires. Participants were excluded if they were younger than 18 years, did not have Portuguese as their first language, or did not have sufficient educational background to adequately understand the study questionnaires.

Prior to participation, all individuals were provided with detailed information about the study objectives and procedures, in accordance with the ethical principles of the Declaration of Helsinki, and electronic informed consent was obtained from all participants. Participation was voluntary, and no financial incentives were offered. Data were collected through an online survey comprising self-report measures assessing exposure to childhood maltreatment, environmental identity, fire-related variables, and sociodemographic characteristics. The survey was administered via a web-based platform and took approximately 20 min to complete. The survey was administered using Jotform, which was configured to prevent repeated submissions by identifying and blocking multiple submissions from the same IP address. To identify inattentive responding, attention-check questions were incorporated throughout the survey, and 112 participants who failed these checks were excluded from the final dataset.

### 2.2. Measures

The Childhood Trauma Questionnaire (CTQ; Bernstein et al., 2003; Matos & Pereira, 2012) is a 28-item retrospective self-report measure assessing experiences of childhood and adolescent maltreatment. The CTQ comprises five subscales: emotional abuse, physical abuse, sexual abuse, emotional neglect, and physical neglect. Items are rated on a 5-point Likert scale ranging from 1 (“never true”) to 5 (“very often true”). Subscale scores are calculated by summing the corresponding items, with higher scores reflecting greater exposure to childhood trauma. In the present study, the CTQ demonstrated satisfactory to excellent internal consistency, with Cronbach’s alpha coefficients of 0.83 for emotional abuse, 0.84 for physical abuse, 0.92 for sexual abuse, 0.87 for emotional neglect, and 0.74 for physical neglect.

Environmental identity was assessed using the Revised Environmental Identity Scale (Revised EID; Clayton et al., 2021; Ferrajão et al., 2024), a 14-item self-report measure rated on a 7-point Likert scale ranging from 1 (“not at all”) to 7 (“true of me”). Although originally conceptualized as a unidimensional measure, subsequent cross-cultural research, including the Portuguese validation study, has supported a two-factor structure comprising Connectedness with Nature and Protection of Nature. Connectedness with Nature reflects individuals’ sense of belonging to and identification with the natural environment, whereas Protection of Nature captures attitudes and values related to environmental stewardship and conservation. Dimension scores were calculated by averaging the corresponding item responses, with higher scores indicating stronger environmental identity. In the present study, the scale demonstrated satisfactory to good internal consistency, with Cronbach’s alpha coefficients of 0.93 for Connectedness with Nature and 0.76 for Protection of Nature.

The Adapted Firesetting Assessment Scale (AFAS; Collins et al., 2022) is a self-report instrument designed to assess fire-related psychological constructs, including fire interest, attitudes toward fire, normalization of fire use, fire safety practices, and identification with fire. The instrument originally comprised 41 items, of which 27 are rated on a 3-point Likert scale ranging from 1 (“Agree”) to 3 (“Disagree”), and 14 are rated on a 4-point Likert scale ranging from 1 (“Very upset/scared”) to 4 (“Excited/Fun”). Several items are reverse-scored prior to analysis. Subsequently, the authors developed a shortened version of the measure comprising 27 items that effectively differentiated individuals with a history of firesetting from non-firesetters. Exploratory factor analysis supported a four-factor structure, yielding the following subscales: Identification with Fire, Poor Fire Safety, Fire as Normal, and Serious Fire Interest (Collins et al., 2022). The total AFAS score is calculated by averaging responses across all completed items, with higher scores indicating more problematic fire-related attitudes and cognitions. Subscale scores are computed using the same procedure, averaging responses across the items comprising each respective subscale.

The suitability of the AFAS for the present community sample was considered in light of the population for which the original measure was developed and evaluated. Although the AFAS was initially developed in the context of firesetting research involving individuals with intellectual disabilities and was subsequently examined in relation to fire setting history, the constructs assessed by the instrument—such as identification with fire, fire-related safety practices, normalization of fire, and serious interest in fire—are not intrinsically restricted to clinical or forensic populations. These constructs may also occur at varying levels in community populations and are therefore theoretically relevant to the present investigation, which focuses on individual differences in self-reported fire-related attitudes and tendencies rather than diagnosed pyromania or verified fire-setting behavior. Nevertheless, we acknowledge that the transfer of an instrument across populations requires empirical examination of its measurement properties rather than assuming that validity established in the original population automatically generalizes to other samples.

As no Portuguese version of the instrument was available, the questionnaire was translated and culturally adapted in accordance with the International Test Commission guidelines (Hernández et al., 2020). Two independent translators, blinded to the study objectives, produced separate Portuguese versions of the scale. Both translators were fluent in English and Portuguese, had expertise in psychometric methods, and possessed extensive knowledge of forensic sciences. The two versions were subsequently compared, revealing a high level of agreement (Cohen’s κ = 0.93). Any discrepancies were reviewed by a panel comprising the translators and members of the research team familiar with the sociocultural context of Portuguese-speaking populations. Following minor cultural and linguistic adaptations, a consensual Portuguese version of the instrument was established.

A pilot study was conducted with a sample whose sociodemographic characteristics closely resembled those of the target population. The sample comprised 27 participants aged between 18 and 73 years, including 18 women and 9 men. The primary objective of the pilot study was to assess the clarity, comprehensibility, and cultural appropriateness of the scale items, as well as to identify any potential issues related to item wording. Participants reported no difficulties in understanding or interpreting the items, and no concerns regarding their relevance or suitability were raised. Consequently, no modifications to the scale were deemed necessary.

The translated scale derived from the cross-cultural adaptation process was administered to the participants. The findings supported the replication of the original four-factor structure while retaining all 27 items and indicated that the measurement model demonstrated satisfactory goodness-of-fit indices in the study sample (χ^2^(121) = 410.94, *p* < 0.01; ratio of chi square to degrees of freedom = 3.40; NFI = 0.99; CFI = 0.99; TLI = 0.98; RMSEA = 0.02; SMSR = 0.02). The reliability of the Identification with Fire scale (α = 0.79), Poor Fire Safety scale (α = 0.73), Fire as Normal scale (α = 0.72), and Serious Fire Interest scale (α = 0.81) was satisfactory to good. These findings provide preliminary evidence of structural validity and internal reliability of the Portuguese AFAS within the present sample. Importantly, however, these analyses do not establish measurement invariance or equivalence of the AFAS between the present community sample and the population in which the original instrument was developed. Therefore, the present study does not interpret absolute AFAS scores as directly comparable with scores obtained in the original population. Rather, AFAS scores are interpreted as indicators of individual differences in fire-related attitudes and tendencies within the present community sample.

Sociodemographic information was collected, including participants’ gender, age, marital status, educational attainment, occupational status, residential setting, and socioeconomic background.

### 2.3. Statistical Methods

Descriptive statistics, including means, standard deviations, and minimum and maximum values, were calculated for all study variables. Bivariate associations among the variables were examined using Pearson’s correlation coefficients, which were interpreted according to conventional thresholds as strong (±0.70–1.00), moderate (±0.30–0.69), or weak (<±0.29). These statistical analyses were conducted using IBM SPSS Statistics for Windows (Version 29).

The hypothesized indirect effects of different types of childhood maltreatment on self-reported attitudes and tendencies toward deliberate fire-setting through environmental identity were examined using a multiple mediation model with bias-corrected and accelerated (BCa) bootstrap confidence intervals (Hayes, 2013). To evaluate the adequacy of the proposed model, structural equation modeling (SEM) was conducted using AMOS version 29. The dataset contained no missing values, as only participants who completed all study measures were included in the analyses.

Specifically, the model assessed: (a) the direct effects of ACE dimensions—emotional abuse, physical abuse, sexual abuse, emotional neglect, and physical neglect—on the overall score of self-reported attitudes and tendencies toward deliberate fire-setting; (b) the indirect effects of these ACE dimensions on fire-setting attitudes and tendencies through the two dimensions of environmental identity, namely connectedness with nature and protection of nature. Model estimation was performed using SEM with the Maximum Likelihood (ML) method, following the recommendations of Hoyle and Smith (1994). All study variables were specified as observed variables.

Model fit was evaluated using established SEM fit indices. Adequate model fit was indicated by: (a) a non-significant chi-square (χ^2^) statistic; (b) Comparative Fit Index (CFI), Normed Fit Index (NFI), and Tucker–Lewis Index (TLI) values greater than 0.95; and (c) Root Mean Square Error of Approximation (RMSEA) and Standardized Root Mean Square Residual (SRMR) values below 0.08. Indirect effects were examined using the bootstrapping approach proposed by Preacher and Hayes (2008), based on 5000 bootstrap resamples. Bias-corrected and accelerated (BCa) 95% confidence intervals (CIs) were estimated for indirect effects. Indirect effects were considered statistically significant when the corresponding confidence interval did not include zero.

Given the conceptual and empirical overlap among the five childhood maltreatment dimensions, additional analyses were conducted to assess potential multicollinearity among the predictors. Variance Inflation Factor (VIF) and Tolerance statistics were examined using multiple linear regression models in IBM SPSS Statistics, with the same predictor sets specified in the structural equation models. Specifically, VIF and Tolerance were assessed for models predicting connectedness with nature and protection of nature from the five childhood maltreatment dimensions, as well as for the model predicting self-reported attitudes and tendencies toward deliberate fire-setting from the five maltreatment dimensions and the two environmental identity dimensions. VIF values below 5 and tolerance values above 0.20 were considered indicative of an absence of potentially problematic multicollinearity.

A multi-group path analysis was conducted to examine whether the structural associations specified in the hypothesized model differed across rural, suburban, and urban participants. This approach allowed the structural parameters to be estimated separately for each residential setting and provided a formal framework for testing whether corresponding regression coefficients were statistically equivalent across groups (Kline, 2023). Because all study variables were represented by composite/observed scores rather than item-level latent factors, the analysis of residential-setting differences was conducted as a multi-group path analysis rather than as a conventional item-level measurement-invariance analysis. The primary objective was therefore to assess whether the hypothesised structural associations varied as a function of residential setting, rather than to evaluate measurement invariance at the item or latent-factor level.

The analysis followed a sequential procedure. First, an unconstrained model was estimated in which all hypothesized structural paths were freely estimated across the three residential groups. The same structural specification was applied to all groups, but no equality restrictions were imposed on the regression coefficients. This model therefore served as the baseline model and allowed each structural association to vary in magnitude and direction across rural, suburban, and urban participants.

Second, a fully constrained model was estimated in which all corresponding structural paths were constrained to equality across the three residential groups. Thus, for each hypothesized association, the regression coefficient was required to be identical across rural, suburban, and urban participants. Because the fully constrained model was nested within the unconstrained model, the two models were compared to determine whether imposing equality restrictions resulted in a statistically meaningful deterioration in model fit. A significant difference in model fit was interpreted as evidence that at least one structural association differed across residential settings, whereas a non-significant difference was considered consistent with structural invariance across groups.

Given the sensitivity of the chi-square difference test to sample size, changes in approximate fit indices were also considered when evaluating the impact of the equality constraints. Specifically, changes in the Comparative Fit Index (ΔCFI), Root Mean Square Error of Approximation (ΔRMSEA), and Standardized Root Mean Square Residual (ΔSRMR) were examined alongside the chi-square difference statistic. A change in CFI of less than 0.01 was considered indicative of negligible deterioration in model fit, consistent with the criterion proposed by Cheung and Rensvold (2002). Changes in RMSEA and SRMR were interpreted according to the complementary criteria proposed by Chen (2007). These indices were considered jointly to distinguish statistically detectable differences from differences that represented a potentially meaningful deterioration in overall model fit.

Third, a targeted multi-group analysis was conducted for the Emotional Abuse → Connectedness with Nature association, motivated by the coefficient reversal observed in the overall sample, whereby the bivariate association was negative but became positive after adjustment for the other maltreatment dimensions. The path was freely estimated across rural, suburban, and urban participants and subsequently constrained to equality across groups. The unconstrained and constrained models were compared to determine whether the association differed by residential setting. Evidence of heterogeneity was inferred from a significant deterioration in model fit following the equality constraint, whereas a non-significant model comparison and negligible changes in approximate fit indices supported cross-group equivalence.

Importantly, group differences were assessed through formal equality-constraint tests rather than by comparing the statistical significance of path coefficients within individual groups. Thus, differences in significance across groups were not, in themselves, interpreted as evidence of structural heterogeneity. Overall, this sequential approach enabled us to distinguish general structural heterogeneity from residential differences in the specific conditional association of interest.

## 3. Results

### 3.1. Descriptive Statistics and Intercorrelations Between Variables

Table 2 summarizes the descriptive statistics and bivariate associations among the study variables, presenting the means, standard deviations, and correlation coefficients. The five types of childhood maltreatment were moderately and positively associated with one another and with self-reported attitudes and tendencies toward deliberate fire-setting, and weakly and negatively associated with both connectedness with nature and protection of nature. Connectedness with nature was moderately positively associated with nature protection and moderately negatively associated with self-reported attitudes and tendencies toward deliberate fire-setting. Protection of nature, in turn, was weakly negatively associated with self-reported attitudes and tendencies toward deliberate fire-setting.

### 3.2. Associations Between Childhood Maltreatment, Environmental Identity, and Attitudes and Tendencies Toward Deliberate Fire-Setting

The direct associations of the five ACE dimensions (emotional abuse, physical abuse, sexual abuse, emotional neglect, and physical neglect) with self-reported attitudes and tendencies toward deliberate fire-setting were examined, along with their indirect associations through two dimensions of environmental identity—connectedness with nature and protection of nature. The proposed model demonstrated excellent goodness-of-fit indices, indicating a strong correspondence between the hypothesized structure and the observed data (χ^2^(5) = 3.18, *p* = 0.67; NFI = 0.99; CFI = 0.99; TLI = 0.99; RMSEA = 0.01; SRMR = 0.01; see Table 3). The model explained 32.4% of the variance in the dependent variable (R^2^ = 0.324).

As shown in Table 3, physical abuse, sexual abuse, emotional neglect, and physical neglect were significantly and directly associated with self-reported attitudes and tendencies toward deliberate fire-setting. Specifically, greater exposure to these forms of childhood maltreatment was associated with higher levels of self-reported attitudes and tendencies toward deliberate fire-setting. Regarding indirect effects, emotional abuse, sexual abuse, emotional neglect, and physical neglect showed significant indirect associations with self-reported attitudes and tendencies toward deliberate fire-setting through connectedness with nature. Specifically, lower levels of emotional abuse and higher levels of sexual abuse, emotional neglect, and physical neglect were associated with lower connectedness with nature, which was, in turn, associated with higher levels of self-reported attitudes and tendencies toward deliberate fire-setting. Additionally, emotional neglect showed a significant indirect association with self-reported attitudes and tendencies toward deliberate fire-setting through protection of nature. Specifically, higher levels of emotional neglect were associated with lower levels of protection of nature, which was, in turn, associated with higher levels of self-reported attitudes and tendencies toward deliberate fire-setting. The remaining indirect associations were not statistically significant. Standardized results are presented in Figure 1.

### 3.3. Multicollinearity and Sensitivity Analysis

The multicollinearity diagnostics did not indicate problematic multicollinearity among the predictors. In the regression models predicting connectedness with nature and protection of nature from the five childhood maltreatment dimensions, Tolerance values ranged from 0.46 to 0.74, whereas VIF values ranged from 1.35 to 2.18. In the model predicting self-reported attitudes and tendencies toward deliberate fire-setting from the five maltreatment dimensions and the two environmental identity dimensions, Tolerance values ranged from 0.45 to 0.73, whereas VIF values ranged from 1.37 to 2.22. All VIF values were below 5, and all Tolerance values exceeded 0.20, indicating that multicollinearity was not sufficiently pronounced to compromise the estimation of the regression coefficients.

To further investigate the unexpected positive association between emotional abuse and connectedness with nature observed in the multivariable model, we conducted a sensitivity analysis to assess the robustness of this association by comparing the bivariate model with the fully adjusted model, including plausible confounders such as age and sex. In the bivariate model, emotional abuse was negatively associated with connectedness with nature (β = −0.12, *p* < 0.01, 95% CI, −0.14, −0.10). However, when physical abuse, sexual abuse, emotional neglect, and physical neglect were simultaneously included in the model, the association between emotional abuse and connectedness with nature changed direction and became positive (β = 0.13, *p* < 0.01, 95% CI, 0.11, 0.15). This coefficient reversal indicates that the positive association observed in the fully adjusted model does not reflect the unadjusted relationship between the two variables, but rather the unique association between emotional abuse and connectedness after accounting for the variance shared with the other maltreatment dimensions.

### 3.4. Differences in Structural Paths Across Residential Settings

A multi-group path analysis was conducted to examine whether the structural associations differed across rural, suburban, and urban residential settings. The unconstrained model (M1), in which structural paths were freely estimated across groups, showed excellent fit to the data, χ^2^(15) = 17.87, *p* = 0.27, CFI = 0.999, RMSEA = 0.006, and SRMR = 0.023. Imposing equality constraints on all structural paths (M2) resulted in a statistically significant increase in the chi-square statistic, Δχ^2^(19) = 61.46, *p* < 0.001. However, given the sensitivity of the chi-square difference test to sample size, changes in approximate fit indices were also considered. The change in CFI was negligible (ΔCFI = −0.002), while the changes in RMSEA (ΔRMSEA = 0.010) and SRMR (ΔSRMR = 0.020) remained below the prespecified thresholds for meaningful deterioration in model fit. Thus, although the chi-square difference test indicated a statistically detectable change in fit, the approximate fit indices did not indicate a substantively meaningful deterioration following the imposition of equality constraints across residential settings.

To further examine whether the Emotional Abuse → Connectedness with Nature association differed across residential settings, a targeted invariance analysis was conducted. In the targeted unconstrained model (M3), this path was freely estimated across groups, whereas in the targeted equality-constrained model (M4), the focal path was constrained to equality while the remaining structural paths were allowed to vary. As shown in Table 4, constraining the focal path did not significantly worsen model fit, Δχ^2^(2) = 1.72, *p* = 0.42. Moreover, the changes in approximate fit indices were negligible (ΔCFI = −0.000, ΔRMSEA = −0.001, and ΔSRMR = 0.002), all remaining well within the prespecified criteria. Taken together, these findings provide evidence that the Emotional Abuse → Connectedness with Nature association is invariant across rural, suburban, and urban residential settings. Accordingly, the coefficient reversal observed in the overall sample does not appear to be attributable to differences in this association across residential contexts.

Given that the multi-group path analysis indicated that the structural associations were largely invariant across rural, suburban, and urban residential settings, a multivariate analysis of variance (MANOVA) was conducted as a complementary analysis to examine whether the groups nonetheless differed in their mean levels across the study variables. Preliminary diagnostic analyses confirmed that the assumptions of normality, linearity, absence of outliers, homogeneity of variance–covariance matrices, and absence of multicollinearity were adequately satisfied, with no meaningful violations observed. Statistically significant differences were identified among the groups on the combined dependent variables, *F*(16, 10174) = 5.98, *p* < 0.001, *η*^2^ = 0.06. Post hoc univariate analyses with Bonferroni-adjusted significance levels (α = 0.05) indicated no statistically significant between-group differences in emotional abuse, emotional neglect, or physical neglect. In contrast, significant differences between groups were observed for physical abuse, sexual abuse, connectedness with nature, protection of nature, and self-reported attitudes and tendencies toward deliberate fire-setting. Effect sizes, as indicated by partial eta squared (*ηp*^2^), were small (Table 5).

As shown in Table 6, scores for physical abuse were significantly higher in the urban subgroup than in all other groups (*p* = 0.02–0.04). Similarly, scores for sexual abuse were significantly higher in the urban subgroup than in the suburban subgroup (*p* = 0.02). Self-reported attitudes and tendencies toward deliberate fire-setting were significantly higher in the urban sub-group than in the suburban sub-group (*p* = 0.001), with no significant differences between the urban and rural sub-groups. In contrast, scores for connectedness with nature and protection of nature were significantly lower in the urban subgroup than in all other groups (*p* = 0.001).

## 4. Discussion

The present study extends the literature on deliberate fire-setting by examining the interplay between childhood maltreatment, connectedness with nature, and nature-protective attitudes within an integrated developmental–ecological framework. To our knowledge, this is the first study to investigate whether connectedness with nature and nature-protective attitudes represent potential indirect pathways linking different forms of childhood maltreatment to self-reported attitudes and tendencies toward deliberate fire-setting, while also examining whether these associations are equivalent across rural, suburban, and urban residential contexts. Furthermore, this research contributes novel empirical evidence from Portugal, a country in which deliberate fire-setting has substantial environmental, social, and economic consequences in the context of a high incidence of wildfires and the predominance of human-caused fires. By addressing an important gap in the literature, the present findings contribute to a more comprehensive understanding of developmental and environmental factors associated with fire-related attitudes and tendencies and provide evidence from a Mediterranean context that has been comparatively overlooked in previous research.

Overall, the findings provide evidence for associations between childhood maltreatment, environmental identity, and self-reported attitudes and tendencies toward deliberate fire-setting. Importantly, these associations were more differentiated than would be expected from a simple cumulative-risk perspective. Physical abuse, sexual abuse, emotional neglect, and physical neglect were directly and positively associated with self-reported attitudes and tendencies toward deliberate fire-setting, indicating that greater exposure to these forms of childhood maltreatment was associated with higher levels of fire-related attitudes and tendencies. In addition, several forms of childhood maltreatment showed significant indirect associations with the outcome through connectedness with nature, while emotional neglect also showed a significant indirect association through protection of nature. Taken together, these findings suggest that the developmental correlates of fire-related attitudes and tendencies may extend beyond established emotional, interpersonal, and behavioral domains to include individuals’ psychological relationships with the natural environment.

### 4.1. Childhood Maltreatment and Deliberate Fire-Setting

The direct associations observed between childhood maltreatment and self-reported attitudes and tendencies toward deliberate fire-setting are broadly consistent with developmental and ecological perspectives emphasizing the relevance of adverse childhood experiences to later emotional and behavioral functioning (Bronfenbrenner & Morris, 2006; Fritzon et al., 2011). Specifically, physical abuse, sexual abuse, emotional neglect, and physical neglect were positively associated with the outcome, suggesting that exposure to these forms of adversity may constitute relevant developmental correlates of fire-related attitudes and tendencies. These findings are consistent with evidence linking childhood maltreatment to difficulties in emotion regulation, behavioral control, interpersonal functioning, and externalizing outcomes (Brereton et al., 2020; Norman et al., 2012; Root et al., 2008; Teicher & Samson, 2016).

Sexual abuse is particularly noteworthy given its direct association with fire-related attitudes and tendencies and its broader relevance across the developmental literature. Childhood sexual abuse has been associated with persistent difficulties in emotion regulation, impulse control, interpersonal functioning, and trauma-related psychopathology (Maniglio, 2009; Norman et al., 2012). Such vulnerabilities may be relevant to the development of maladaptive behavioral tendencies, including deliberate fire-setting, particularly when fire-related activities become incorporated into broader patterns of emotional dysregulation or maladaptive coping (Fritzon et al., 2011; Johnston & Tyler, 2025; Wehner et al., 2022). Within the Motivational Theory of Arson and related developmental formulations, fire-setting may emerge from the interaction between individual vulnerabilities and situational or contextual factors rather than from a single underlying cause (Fritzon et al., 2011). The present findings are compatible with this perspective by indicating that several forms of childhood adversity are associated with fire-related attitudes and tendencies.

The findings concerning neglect further underscore the potential relevance of developmental deprivation. Both emotional and physical neglect were directly associated with higher levels of self-reported attitudes and tendencies toward deliberate fire-setting. Neglect has consistently been associated with impairments in emotional development, social competence, attachment, and the capacity to recognize and regulate emotional states (Norman et al., 2012; Teicher & Samson, 2016). Such developmental disruptions may contribute to maladaptive externalizing tendencies by compromising adaptive coping and emotional self-regulation. In the context of deliberate fire-setting, these processes may be particularly relevant when individuals use externalizing behaviors as a means of regulating distress, seeking stimulation, or responding to interpersonal difficulties (Fritzon et al., 2011; Root et al., 2008).

Physical abuse was also directly associated with higher levels of self-reported attitudes and tendencies toward deliberate fire-setting. This finding is consistent with research linking childhood physical abuse to aggression, hostile attribution biases, behavioral disinhibition, and difficulties in emotion regulation (Gilbert et al., 2009; Norman et al., 2012). These developmental characteristics may increase vulnerability to destructive or externalizing behavioral tendencies, although the present findings do not establish that physical abuse specifically causes deliberate fire-setting. Rather, physical abuse may represent one component of a broader developmental risk profile associated with maladaptive behavioral and emotional functioning.

Importantly, the multigroup analyses indicated that the structural associations among childhood maltreatment, environmental identity, and self-reported attitudes and tendencies toward deliberate fire-setting were largely invariant across rural, suburban, and urban residential settings. Although constraining the structural paths to equality produced a statistically significant chi-square difference, the changes in CFI, RMSEA, and SRMR remained below the prespecified criteria for meaningful deterioration in model fit. Thus, the statistically significant chi-square difference should not be interpreted as evidence of substantively different developmental pathways across residential contexts. Instead, the pattern of approximate fit indicates that the associations were broadly comparable across groups. This finding is important because it suggests that residential context may be more relevant to differences in the levels of particular variables than to the fundamental structural relationships linking childhood maltreatment, environmental identity, and fire-related attitudes.

The absence of substantial structural heterogeneity across residential settings does not imply that context is irrelevant. Rather, consistent with ecological models, residential environments may influence the developmental experiences and environmental opportunities available to individuals without necessarily modifying the associations among the constructs examined (Bronfenbrenner & Morris, 2006). Accordingly, the present findings support an ecological interpretation in which developmental adversity and environmental characteristics are embedded within broader contexts, while also indicating that the pathways examined here may be relatively robust across different residential settings.

### 4.2. Environmental Identity as a Mechanism Linking Childhood Maltreatment to Deliberate Fire-Setting

The present findings provide novel evidence that environmental identity may represent a potential psychosocial pathway linking childhood maltreatment to self-reported attitudes and tendencies toward deliberate fire-setting. Although previous models of deliberate fire-setting have primarily emphasized psychopathological, emotional, interpersonal, and criminogenic processes (Fritzon et al., 2011; Wehner et al., 2022), the current results suggest that individuals’ psychological relationships with the natural environment may constitute an additional correlate within a developmental framework. Specifically, emotional abuse, sexual abuse, emotional neglect, and physical neglect showed significant indirect associations with fire-related attitudes and tendencies through connectedness with nature. These findings suggest that childhood adversity may be associated not only with interpersonal and intrapersonal functioning but also with individuals’ psychological relationships with the broader ecological environment (Schultz, 2001).

Connectedness with nature reflects the extent to which individuals experience themselves as emotionally and psychologically connected to the natural world and has been associated with environmental concern, environmental identity, and pro-environmental behavior (Mayer & Frantz, 2004; Nisbet et al., 2009; Whitburn et al., 2020). Conversely, lower connectedness with nature may reflect greater psychological distance from the natural environment and weaker incorporation of nature into the self-concept (Clayton et al., 2021; Schultz & Tabanico, 2007). Within this framework, the present findings suggest that lower connectedness with nature is associated with higher levels of self-reported attitudes and tendencies toward deliberate fire-setting. One possible interpretation is that individuals who experience a weaker psychological bond with the natural environment may have fewer affective or identity-based reasons to perceive damage to natural environments as personally relevant (Clayton, 2003; Schultz & Tabanico, 2007). However, this interpretation remains tentative, and the present data cannot establish that connectedness with nature causally influences fire-setting attitudes or behavior.

The indirect associations involving connectedness with nature were observed for several forms of childhood maltreatment, suggesting that this pathway may be broader than any single form of developmental adversity. Sexual abuse, emotional neglect, and physical neglect were associated with lower connectedness with nature, which was in turn associated with higher levels of fire-related attitudes and tendencies. These findings may be understood in light of evidence that adverse childhood experiences can disrupt attachment, emotional regulation, interpersonal trust, and feelings of belonging (Cicchetti & Toth, 2005; Norman et al., 2012; Teicher & Samson, 2016). Because connectedness with nature involves feelings of relatedness and psychological inclusion within the natural world (Mayer & Frantz, 2004; Nisbet et al., 2009), developmental disruptions in relational functioning may potentially extend beyond interpersonal relationships to broader forms of affiliation. This interpretation is consistent with theoretical perspectives proposing that environmental identity develops partly through processes of belonging, identification, and affective connection (Clayton et al., 2021; Schultz, 2001).

The finding that connectedness with nature was relevant across residential contexts is particularly important. Although rural environments generally provide greater physical exposure to natural landscapes, physical proximity to nature does not necessarily translate into stronger psychological connectedness with nature. Research suggests that the quality, frequency, and subjective meaning of interactions with nature may be more important than geographical proximity alone (Anderson & Krettenauer, 2021; Hinds & Sparks, 2008; Soga & Gaston, 2016). Accordingly, the present findings caution against assuming that rural residence necessarily confers stronger environmental identity. Instead, meaningful and emotionally salient interactions with nature may be particularly relevant to the development of connectedness with nature, irrespective of residential setting.

The association between emotional abuse and connectedness with nature requires particular caution in interpretation. At the bivariate level, emotional abuse was negatively associated with connectedness with nature. However, after physical abuse, sexual abuse, emotional neglect, and physical neglect were simultaneously entered into the model, the association reversed direction and became positive. Such a reversal in the direction of an association following statistical adjustment can occur when predictors share variance and is consistent with a potential suppression effect (MacKinnon et al., 2000). This coefficient reversal indicates that the positive association observed in the fully adjusted model does not represent the unadjusted relationship between emotional abuse and connectedness with nature. Rather, it reflects the conditional association between emotional abuse and connectedness with nature after accounting for the variance shared with the other maltreatment dimensions (Aloe, 2015).

Importantly, the multicollinearity diagnostics indicated that the correlations among the maltreatment dimensions did not reach levels typically considered indicative of problematic multicollinearity, as evidenced by acceptable tolerance and VIF values. However, such diagnostics should be interpreted cautiously, as no single VIF threshold provides a definitive indication of problematic multicollinearity across all regression contexts (O’Brien, 2007). Nevertheless, the observed change in the magnitude and direction of the emotional abuse coefficient suggests that shared variance among the maltreatment dimensions may contribute to a suppression effect, whereby controlling for correlated predictors can alter the magnitude and direction of a predictor’s association with the outcome (Conger, 1974). Accordingly, the positive conditional association should be interpreted cautiously as a statistical association that emerges after controlling for overlapping maltreatment dimensions. This interpretation is further supported by the contrast between the negative bivariate association and the positive coefficient obtained in the fully adjusted model. Replication using longitudinal and alternative statistical approaches will be important for determining whether this coefficient reversal is robust and for clarifying its substantive meaning.

The targeted multigroup analysis provides additional support for this interpretation. The Emotional Abuse → Connectedness with Nature pathway did not differ significantly across rural, suburban, and urban settings, and constraining this path to equality produced negligible changes in the approximate fit indices. Thus, the coefficient reversal observed in the overall adjusted model does not appear to be attributable to differences in this association across residential contexts. Instead, the positive conditional association appears to be broadly comparable across groups. This finding reinforces the distinction between statistical invariance of structural relationships and differences in the mean levels of the constructs across residential settings.

The indirect effect through protection of nature provides complementary evidence that different dimensions of environmental identity may operate through partially distinct pathways. Emotional neglect showed a significant indirect association with self-reported attitudes and tendencies toward deliberate fire-setting through protection of nature, such that higher emotional neglect was associated with lower protection of nature, which was subsequently associated with higher levels of fire-related attitudes and tendencies. This finding is conceptually consistent with literature indicating that environmental concern and pro-environmental orientations are associated with environmental identity, moral concern, and connectedness with nature (Clayton & Opotow, 2003; Clayton et al., 2021; Stern, 2000; Whitburn et al., 2020).

The specificity of the emotional-neglect pathway through protection of nature is theoretically noteworthy. Whereas connectedness with nature was implicated in indirect associations involving several forms of maltreatment, protection of nature emerged specifically in the indirect association involving emotional neglect. This distinction suggests that feeling psychologically connected to nature and endorsing attitudes oriented toward protecting nature may represent related but non-equivalent dimensions of environmental identity. Connectedness with nature primarily concerns an individual’s affective and cognitive relationship with the natural world, whereas protection of nature more directly reflects an orientation toward environmental preservation and responsibility. Previous research similarly suggests that different dimensions of the human–nature relationship, although correlated, may have distinct antecedents and behavioral correlates (Clayton et al., 2021; Mayer & Frantz, 2004; Whitburn et al., 2020).

The association between emotional neglect and lower protection of nature may also be considered in relation to broader developmental processes. Emotional neglect involves persistent deficits in emotional responsiveness and support and has been associated with difficulties in attachment, empathy, social competence, and emotional development (Cicchetti & Toth, 2005; Teicher & Samson, 2016). Because environmental concern and protective orientations may partly depend on affective and empathic processes, developmental experiences characterized by insufficient emotional responsiveness may be relevant to the development of weaker protective orientations toward the natural environment. However, this interpretation remains speculative, and longitudinal evidence is required to determine whether emotional neglect precedes changes in environmental identity and whether these changes subsequently contribute to fire-related attitudes or behaviors.

The residential group comparisons provide an important complementary perspective on these structural findings. Although the structural relationships were largely invariant across rural, suburban, and urban settings, significant mean-level differences emerged across groups. Urban participants reported lower levels of connectedness with nature and protection of nature than both rural and suburban participants. These findings are consistent with previous research suggesting that residential context is associated with differences in connectedness with nature and environmental orientations (Anderson & Krettenauer, 2021; Hinds & Sparks, 2008; Macias-Zambrano et al., 2024; Sierra-Barón et al., 2023). Urban contexts may provide fewer opportunities for sustained or meaningful engagement with natural environments, potentially limiting opportunities for the development of affective bonds with nature (Soga & Gaston, 2016). Nevertheless, urban residence should not be conceptualized as synonymous with weak environmental identity. Rather, the quality and meaning of interactions with nature may be more consequential than physical proximity alone (Anderson & Krettenauer, 2021; Hinds & Sparks, 2008).

The present findings therefore suggest an important distinction between contextual differences in levels and contextual differences in processes. Urban participants reported lower levels of connectedness with nature and protection of nature, yet the associations linking childhood maltreatment to these dimensions and, in turn, to fire-related attitudes and tendencies were largely comparable across residential settings. From an ecological perspective, this pattern suggests that residential environments may be associated with the extent to which individuals experience particular developmental and environmental characteristics without necessarily altering the fundamental relationships among those characteristics (Bronfenbrenner & Morris, 2006). Future research should therefore move beyond broad rural–suburban–urban classifications and examine more proximal environmental characteristics, including the quality and frequency of nature experiences, access to green spaces, neighborhood social cohesion, socioeconomic conditions, and perceived environmental stress.

The MANOVA findings also indicated that urban participants reported higher levels of physical abuse than both rural and suburban participants and higher levels of sexual abuse than suburban participants. No significant residential differences emerged for emotional abuse, emotional neglect, or physical neglect. These findings are broadly compatible with evidence indicating that exposure to childhood maltreatment may vary across neighborhood and socioeconomic contexts (Coulton et al., 2007; Finkelhor et al., 2015). However, these differences should not be interpreted as demonstrating that urban residence itself increases the risk of maltreatment. Residential setting is likely to represent a marker for a broader constellation of social, economic, and neighborhood characteristics, and the present cross-sectional design does not allow these mechanisms to be disentangled.

Urban participants also reported higher levels of self-reported attitudes and tendencies toward deliberate fire-setting than suburban participants, whereas no significant difference emerged between urban and rural participants. This pattern does not support a straightforward urban–rural gradient. Instead, it suggests a more nuanced contextual pattern in which urban and suburban participants differed, whereas urban and rural participants did not. Moreover, the small effect sizes observed in the MANOVA indicate that these differences, although statistically significant, were modest in magnitude. This distinction is particularly important when interpreting group differences in large samples, where relatively small effects may reach statistical significance.

From an ecological perspective, several contextual characteristics may plausibly contribute to differences in fire-related attitudes and tendencies, including environmental stress, neighborhood characteristics, opportunities for contact with nature, and social cohesion (Bronfenbrenner & Morris, 2006; Evans, 2003; Kuo, 2015). Urban environments have also been associated with greater exposure to environmental stressors and reduced opportunities for restorative contact with nature (Bratman et al., 2019; Hartig et al., 2014). Such contextual factors may be relevant to psychological well-being and environmental identity, but the present findings do not permit conclusions regarding their causal contribution to fire-related attitudes. Indeed, the substantial structural invariance observed across residential contexts indicates that contextual characteristics may be more relevant to explaining differences in the levels of individual variables than to modifying the associations among childhood maltreatment, environmental identity, and fire-related tendencies.

Taken together, the present findings support an integrated developmental–ecological account of self-reported attitudes and tendencies toward deliberate fire-setting. Childhood maltreatment was associated with higher levels of fire-related attitudes and tendencies both directly and, for several maltreatment dimensions, indirectly through environmental identity. Connectedness with nature emerged as a relatively broad indirect pathway, whereas protection of nature showed a more specific association involving emotional neglect. At the same time, the structural relationships were largely invariant across rural, suburban, and urban residential settings, despite statistically significant but generally small differences in the mean levels of several study variables. These findings suggest that residential context may be associated with differences in the developmental and environmental conditions in which individuals are embedded without necessarily altering the fundamental associations among childhood adversity, environmental identity, and fire-related attitudes.

### 4.3. Limitations

Several limitations should be considered when interpreting the present findings. First, the cross-sectional design limits causal inferences regarding the relationships among childhood maltreatment, environmental identity, and self-reported attitudes and tendencies toward deliberate fire-setting. Although the indirect effects are consistent with the proposed developmental–ecological model, they should be interpreted as statistical associations rather than evidence of temporal or causal mechanisms. Longitudinal studies are needed to clarify the temporal ordering of these associations and to determine whether environmental identity functions as a developmental pathway linking childhood adversity to later fire-related tendencies. Second, reliance on retrospective self-report measures may introduce recall bias, social desirability effects, and underreporting, particularly for sensitive experiences such as maltreatment and fire-setting tendencies. Third, the convenience sample of Portuguese adults limits the generalizability of the findings across populations and cultural contexts. Although Portugal represents a relevant setting given the prevalence of human-caused wildfires, replication in diverse ecological contexts is needed to assess the robustness of these associations. Additionally, the smaller rural subgroup requires cautious interpretation of context-specific effects. Fourth, deliberate fire-setting was assessed using the AFAS, a self-report measure originally developed and evaluated in a population that differs from the present community sample. Although the four-factor structure and internal consistency were satisfactory in the current sample, these findings provide evidence of psychometric performance within this sample but do not establish measurement invariance with the original population. Accordingly, AFAS scores should not be assumed to be directly comparable across populations or interpreted as indicators of actual fire-setting, arson, or pyromania. Future research should examine cross-population measurement invariance and further establish convergent and discriminant validity against behavioral and clinical indicators of firesetting. Finally, the study examined environmental identity as a potential mechanism but did not consider other relevant factors, including emotion regulation, empathy, moral disengagement, and situational opportunities. Future research should incorporate these variables into broader developmental–ecological models of deliberate fire-setting.

## 5. Conclusions

The present study provides novel evidence that childhood maltreatment and individuals’ relationships with the natural environment are associated with self-reported attitudes and tendencies toward deliberate fire-setting. The findings indicate that several forms of childhood maltreatment were directly associated with higher levels of fire-related attitudes and tendencies, while connectedness with nature emerged as a potential indirect pathway linking multiple forms of maltreatment to the outcome and protection of nature that showed a more specific indirect association involving emotional neglect. Importantly, these findings should be interpreted as evidence of statistical associations rather than causal or mechanistic effects, particularly given the cross-sectional and self-report nature of the data. The largely invariant structural relationships across rural, suburban, and urban contexts further suggest that the pathways examined may be relatively robust across residential settings, even though meaningful differences in the levels of several constructs were observed between groups. Overall, the findings support the value of broadening developmental accounts of deliberate fire-setting to consider the psychological relationship with the natural environment alongside established emotional, interpersonal, and behavioral risk factors. Future longitudinal and multi-method research should test the temporal ordering and replicability of these associations, examine more proximal environmental and developmental characteristics, and determine whether environmental identity represents a meaningful target for prevention and early intervention efforts addressing fire-related risk.

## Figures and Tables

**Figure 1:**
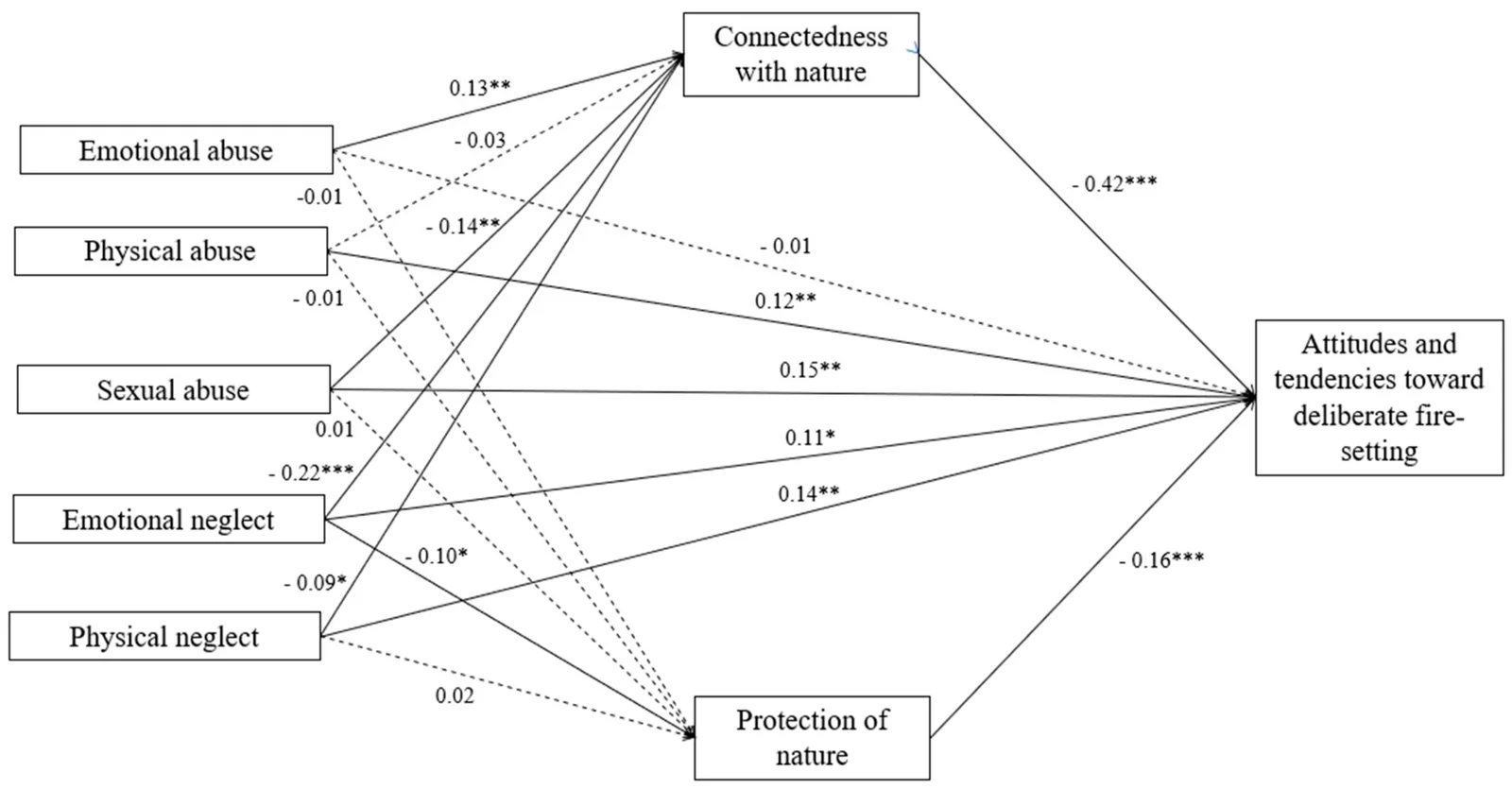
Model of direct and indirect associations between dimensions of childhood maltreatment and attitudes and tendencies toward deliberate fire-setting through environmental identity. *Note.* Unidirectional arrows depict hypothesized directional links. Standardized maximum likelihood parameters are used. Bold line estimates are statistically significant, whereas dashed lines are non-significant. *N* = 5077; * *p* < 0.05, ** *p* < 0.01, *** *p* < 0.001.

**Table 1:** Sample demographic characteristics.

Variables	*n*	%
**Sex**		
Female	3537	69.7
Male	1540	30.3
**Civil status**		
Single	2945	58.0
Married or cohabiting	1767	34.8
Divorced	294	5.8
Widowed	71	1.4
**Education**		
Less than nine years of education	103	2.0
Nine years of education	247	4.9
12 years of education	2256	44.4
Higher education	2471	48.7
**Professional status**		
Student	1668	32.9
Working student	590	11.6
Employee	2361	46.5
Unemployed	245	4.8
Retired	213	4.2
**Residential setting**		
Rural	511	10.1
Suburban	1486	29.3
Urban	3080	60.6
**Socioeconomic status**		
Low	351	6.9
Medium	3628	71.4
Medium-high	1009	19.9
High	89	1.8

**Table 2:** Descriptive statistical results and correlation matrix between variables.

Variable	M (SD)	1	2	3	4	5	6	7	8
1 Emotional abuse	8.60 (4.40)	-	0.64 ***	0.54 ***	0.53 ***	0.41 ***	−0.12 ***	−0.05 ***	0.30 ***
2 Physical abuse	6.88 (3.37)		-	0.57 ***	0.39 ***	0.46 ***	−0.15 ***	−0.02	0.36 ***
3 Sexual abuse	6.60 (3.84)			-	0.31 ***	0.40 ***	−0.20 ***	−0.02	0.37 ***
4 Emotional neglect	10.05 (4.74)				-	0.30 ***	−0.24 ***	−0.10 ***	0.32 ***
5 Physical neglect	10.30 (2.30)					-	−0.17 ***	−0.01	0.35 ***
6 Connectedness with nature	5.70 (1.25)						-	0.69 ***	−0.41 ***
7 Protection of nature	4.50 (1.42)							-	−0.15 ***
8 Attitudes and tendencies toward deliberate fire-setting	1.50 (0.29)								-

*Note.* *** *p* < 0.001.

**Table 3:** Bootstrapped point estimates for direct, indirect and total effects and 95% confidence intervals for predicting self-reported attitudes and tendencies toward deliberate fire-setting by adverse childhood experiences through environmental identity dimensions.

Variable	Effect	Standardized Estimates	95% Bootstrap Confidence Intervals (Lower; Upper)
Emotional abuse	Direct	−0.01	−0.03; 0.01
	Indirect through connectedness with nature	−0.05	−0.08; −0.03
	Indirect through protection of nature	0.00	−0.01; 0.01
	Total	−0.06	−0.08; −0.04
Physical abuse	Direct	0.12	0.09; 0.15
	Indirect through connectedness with nature	0.01	0.00; 0.02
	Indirect through protection of nature	0.00	−0.01; 0.01
	Total	0.13	0.11; 0.15
Sexual abuse	Direct	0.15	0.11; 0.19
	Indirect through connectedness with nature	0.06	0.03; 0.09
	Indirect through protection of nature	0.00	−0.02; 0.02
	Total	0.21	0.17; 0.25
Emotional neglect	Direct	0.11	0.10; 0.12
	Indirect through connectedness with nature	0.10	0.08; 0.12
	Indirect through protection of nature	0.02	0.01; 0.03
	Total	0.23	0.20; 0.26
Physical neglect	Direct	0.14	0.11; 0.17
	Indirect through connectedness with nature	0.04	0.02; 0.06
	Indirect through protection of nature	0.00	−0.01; 0.01
	Total	0.18	0.15; 0.21

*Note*. CI = confidence intervals; confidence intervals that do not include 0 (null association) are significant.

**Table 4:** Multi-group path analysis results by residential settings.

Model	Number of Parameters	χ^2^	df	*p*	χ^2^/df	CFI	RMSEA	SRMR
M1. Unconstrained multi-group	117	17.87	15	0.27	1.19	0.999	0.006	0.023
M2. Fully constrained multi-group	98	79.33	34	0.001	2.33	0.997	0.016	0.043
Model Comparison. M2 vs. M1	−19	61.46	19	0.001	1.14	−0.002	0.010	0.020
M3. Targeted unconstrained	99	78.85	33	0.001	2.39	0.997	0.017	0.044
Model Comparison. M3 vs. M1	−18	60.98	18	0.001	1.20	0.002	0.011	0.021
M4. Targeted equality-constrained	115	19.59	17	0.30	1.15	0.999	0.005	0.025
Model Comparison. M4 vs. M1	−2	1.72	2	0.42	−0.04	0.000	−0.001	0.002

*Note*. Models for the invariance of structural paths across residential settings. χ^2^ = chi-square statistic; df = degrees of freedom; *p* = *p*-value; χ^2^/df = ratio of chi square to degrees of freedom; CFI = Comparative Fit Index; RMSEA = Root Mean Square Error of Approximation; SRMR = Standardized Root Mean Square Residual.

**Table 5:** Tests of between-subjects effects.

Dependent Variables	Mean Square	df	F	*p*	Partial Eta Squared
Emotional abuse	12.60	2	0.65	0.52	0.000
Physical abuse	64.25	2	5.66	0.004	0.002
Sexual abuse	56.68	2	3.86	0.02	0.002
Emotional neglect	62.90	2	2.80	0.06	0.001
Physical neglect	15.26	2	2.88	0.06	0.001
Connectedness with nature	27.50	2	17.60	0.001	0.007
Protection of nature	37.49	2	18.85	0.001	0.007
Attitudes and tendencies toward deliberate fire-setting	0.90	2	9.48	0.01	0.004

**Table 6:** Means and standard deviations of residential contexts on study variables.

Variables	Rural			Suburban			Urban		
	M	SD	95% CI	M	SD	95% CI	M	SD	95% CI
Emotional abuse	8.72	4.42	8.34; 9.10	8.50	4.33	8.28; 8.72	8.63	4.44	8.48; 8.79
Physical abuse	6.61	3.03	6.35; 6.87	6.71	3.25	6.55; 6.88	7.01	3.48	6.88; 7.13
Sexual abuse	6.56	3.78	6.23; 6.89	6.38	3.61	6.20; 6.57	6.72	3.95	6.58; 6.86
Emotional neglect	10.52	4.73	10.11; 10.93	10.05	4.53	9.81; 10.28	9.98	4.84	9.81; 10.15
Physical neglect	10.51	2.31	10.31; 10.71	10.23	2.29	10.11; 10.35	10.30	2.31	10.22; 10.38
Connectedness with nature	5.91	1.21	5.81; 6.02	5.78	1.15	5.73; 5.84	5.62	1.30	5.57; 5.66
Protection of nature	4.72	1.38	4.60; 4.84	4.63	1.33	4.56; 4.69	4.41	1.45	4.36; 4.46
Attitudes and tendencies toward deliberate fire-setting	1.50	0.31	1.47; 1.53	1.47	0.30	1.46; 1.49	1.51	0.31	1.50; 1.53

## Data Availability

The data presented in this study are available on request from the corresponding author due to data protection regulations.

## Abbreviation

The following abbreviation is used in this manuscript:

M-TTAF	Multi-Trajectory Theory of Adult Firesetting
